# Variance in Calvin–Benson cycle intermediate levels between closely related species in the tomato clade

**DOI:** 10.1093/jxb/erag207

**Published:** 2026-05-02

**Authors:** Vittoria Clapero, Stéphanie Arrivault, Mark Stitt

**Affiliations:** Max Planck Institute of Molecular Plant Physiology, Am Muehlenberg 1, D-14476 Potsdam-Golm, Germany; Max Planck Institute of Molecular Plant Physiology, Am Muehlenberg 1, D-14476 Potsdam-Golm, Germany; Max Planck Institute of Molecular Plant Physiology, Am Muehlenberg 1, D-14476 Potsdam-Golm, Germany; University of Illinois Urbana-Champaign, USA

**Keywords:** Calvin–Benson cycle, inter-species variance, intermediate levels, metabolite profiling, *Solanum*, *Solanum pennelli*, tomato clade

## Abstract

Published studies have reported species variance between profiles of Calvin–Benson cycle (CBC) intermediates, not only between C_4_ species and C_3_ species, but also within C_3_ species. It was proposed that this variance reflects lineage-dependent changes in the balance between different reactions, or poising, of the CBC. These earlier studies investigated phylogenetically unrelated C_3_ species. In the current study, CBC intermediates were profiled in five closely related species from *Solanum* sect. *lycopersicon* subsect. *Lycopersicum*. Levels of individual CBC intermediates showed many significant differences. In a principal component analysis, whilst three species (*Solanum lycopersicum*, *Solanum*  *cheesmaniae*, and *Solanum neorickii*) overlapped, *Solanum pimpinellifolium* and especially *Solanum pennellii* grouped separately, and were at opposing ends of the distribution. When combined with published data, whilst the separation between *Solanum* species was retained, they formed a group that was separated from five other C_3_ species, as well as two C_4_ species. It is discussed that the observed variation in CBC metabolites profiles within *Solanum*, together with their separation from other C_3_ species, supports the idea that CBC evolution is shaped by phylogenetic relatedness and, by implication, lineage-specific adaptation.

## Teaser

Variance of intermediate levels points to poising of the Calvin–Benson cycle varying between closely related species in the tomato clade *Solanum* sect. *lycopersicon* subsect. *Lycopersicum*

## Introduction

Since the Calvin–Benson cycle (CBC) evolved in cyanobacteria ∼2 billion years ago (Rasmussen *et al*., 2008), there have been large changes in the conditions under which it operates. Over geological time, there has been a dramatic decline in atmospheric CO_2_ and increase in atmospheric O_2_, including in the last 30 million years, when parallel evolution of C_4_ photosynthesis in >60 lineages coincided with a decline of atmospheric CO_2_ from ∼1000 ppm to <300 ppm (Sage *et al*., 2012; Raven *et al*., 2017). Species that did not evolve C_4_ photosynthesis, termed C_3_ species, representing ∼90% of existing terrestrial plant species (Silvera *et al*., 2010; Sage, 2017), will also have been subject to massive selective pressure by falling CO_2_ as well as its subsequent rise. Pressure will also have been exerted by further environmental factors such as light, temperature, and water and nutrient availability (Raven *et al*., 2017).

There is substantial variation in photosynthetic rate between terrestrial C_3_ species (Evans, 1989; Wullschleger, 1993), including between phylogenetically related species (Galmés *et al*., 2014a, b; Kromdijk and McCormick, 2022) and within species (Driever *et al*., 2014; Flood, 2019). Contributing factors include differing rates of electron transport and carboxylation (Wullschleger, 1993), leaf nitrogen content, nitrogen use efficiency (Field and Mooney, 1986; Evans, 1989; Hikosaka, 2010), stomatal responses (Lawson and Vialet-Chabrand, 2019), and investment strategies in short- and long-lived leaves (Wright *et al*., 2004; Donovan *et al*., 2011). At the level of individual steps, it is well established that Rubisco kinetics vary, even between closely related species (Yeoh *et al*., 1980; Carmo-Silva *et al*., 2010, 2015; Galmés *et al*., 2014a; Orr *et al*., 2016; Prins *et al*., 2016; Hermida-Carrera *et al*., 2017; Bouvier *et al*., 2021). C_3_ species differ in how Rubisco is regulated by low molecular weight inhibitors (Servaites *et al*., 1986; Moore *et al*., 1993; Charlet *et al*., 1997; Parry *et al*., 2008). Regulation by CP12, a small protein that interacts with NADP-glyceraldehyde-3-phosphate and phosphoribulokinase (PRK), varies between species (Howard *et al*, 2011). Recent studies in crops have uncovered variance in Rubisco capacity (*V*_cmax_) (Acevedo-Siaca *et al*., 2020, 2021), heat tolerance of Rubisco activase (Scafaro *et al*., 2019), steady-state and transient rates of photosynthesis (Pignon *et al*., 2021), heat stability of PSI (Posch *et al*., 2022), and cold tolerance (Ortiz *et al*., 2017). Based on evolutionary modelling, Zhu *et al*. (2007) proposed that current-day terrestrial plants may not yet have fully rebalanced the CBC after the low-CO_2_ and low-temperature conditions they experienced in recent ice ages.

In recent years, the detection and mapping of allelic variance have provided an important and unbiased approach to studying variation in photosynthesis (Adachi *et al*., 2019; Faralli and Lawson, 2020; Kromdijk and McCormick, 2022; Sakoda *et al*., 2022; Sharwood *et al*., 2022). Metabolite profiling provides another unbiased approach to detect inter-specific variation, specifically in CBC operation. The underlying assumption is that changes in the relative levels of pathway intermediates reflect changes in the balance between enzymatic steps, or poising, in the pathway. Changes in poising are detected, regardless of whether they are due to changes in enzyme abundance, enzyme properties, or regulatory loops. LC-MS/MS and stable isotope standards allow quantitative profiling of most CBC intermediates (Arrivault *et al*., 2009, 2015, 2019). A diverse set of C_3_ species including the dicots *Arabidopsis thaliana*, *Nicotiana tabacum* (tobacco), *Manihot esculenta* (cassava), and *Flaveria* spp. and the monocots *Oryza sativa* (rice) and *Triticum aestivum* (wheat) showed significantly differing levels of many CBC metabolites (Arrivault *et al*., 2019; Borghi *et al*., 2019, 2022; Stitt *et al*., 2021; Clapero *et al*., 2024). When displayed using principal component (PC) analysis, the changes distinguished one C_3_ species from another. These results were obtained for plants grown in moderately limiting irradiance and non-stressful conditions, and metabolites were profiled in material harvested in growth conditions. Whilst environment influences intermediate levels, the cross-species variance was interpreted as involving the impact of genotype or at least a genotype×environment interaction. The contribution of genotype was underlined by the finding that *A. thaliana* and *O. sativa* showed consistently different levels of CBC intermediates across a range of harvest irradiances (Borghi *et al*., 2019). These observations led to the proposal that this variability arises from continual selection on the CBC, and that this occurs in a lineage-dependent manner, with different lineages following different adaptation trajectories (Arrivault *et al*., 2019; Stitt *et al*., 2021).

In previous studies, species were chosen based on their role as model organisms or important crops, and were phylogenetically widely separated. A set of *Flaveria* C_3_ species, C_3_–C_4_ intermediate species, and C_4_ species was studied to gain insights into the evolution of C_4_ photosynthesis (Borghi *et al*., 2022), but did not provide information about variation between phylogenetically related C_3_ species. The *Solanum* genera, with ∼1500 species, is one of the largest genera of angiosperms. The tomato clade *Solanum* sect. *lycopersicon* subsect. *Lycopersicum* includes the cultivated tomato *Solanum lycopersicum* and 12 wild species (Peralta *et al*., 2008; Knapp and Peralta, 2016; Supplementary Fig. S1A). All members have the same number of chromosomes, high genomic synteny, and are crossable to some degree (Bedinger *et al*., 2011; Knapp and Peralta, 2016). They occur naturally along a 3000 km north–south range of the western Andes and on the Galapagos islands on sites with dry and often montane environments, except *S. pimpinellifolium* which trends towards coastal sites (Peralta *et al*., 2008; Supplementary Fig. S1B).

We have profiled CBC intermediates in five species from the *Solanum* sect. *lycopersicon* (Supplementary Fig. S1A)*: S. lycopersicum*, *S. pimpinellifolium*, *S. cheesmaniae*, *S. neorickii*, and *S. pennelli*. The aims were to investigate if CBC intermediate profiles vary between closely related species, ask if the variance relates to phylogeny, and compare their profiles with those previously reported for phylogenetically distinct species.

## Materials and methods

### Plant growth and harvest

*Solanum lycopersicum* (var. ‘Moneymaker’), *S. pimpinellifolium*, *S. cheesmaniae*, *S. neorickii*, and *S. pennellii* seeds were provided by Saleh Alseek. Seeds were germinated on 2% v/v sucrose agar and grown in a 16 h day/8 h night (irradiance 590 µmol photons m^−2^ s^−1^, 23 °C/20 °C, 60% relative humidity). The apical leaf blade of the third fully expanded leaf was harvested by detaching the leaf and rapidly transferring it under ambient light without shading or turning into a large container of liquid nitrogen. Although quenching was performed in ambient light, we did not measure the speed of leaf freezing (see Xu *et al*., 2024) and cannot exclude slight changes in metabolite levels during cooling. Nevertheless, even if that was the case, it would have affected all samples in the same manner and also those from previous studies that are used here for comparison. Therefore, conditions for cross-compatibility are ensured. For each sample, apical leaves from two plants were randomly pooled prior to grinding and further processing. The number of replicate samples was S. *lycopersicum n*=6, *S. pimpinellifolium n*=6, *S*. *cheesmaniae n*=5, *S. neorickii n*=6, and *S. pennellii n*=7. Each replicate was with leaves from different plants.

### Metabolite extraction and analysis

Frozen leaf material was homogenized with an oscillating ball mill (Retsch, Haan, Germany; https://www.retsch.com) and metabolites were extracted and quantified by LC–MS/MS as in Arrivault *et al*. (2009, 2019). Stable-isotope-labelled internal standards were added to correct for matrix effects (Arrivault *et al*., 2015). 3-Phosphoglycerate (3PGA) was determined enzymatically in freshly prepared trichloroacetic acid extracts (Jelitto *et al*., 1992) using a spectrophotometer (Shimadzu, Kyoto, Japan; www.shimadzu.de).

### Statistical analysis

Statistical analysis (ANOVA and Tukey’s test) was performed in GraphPad Prism version 10.2.2. The *z*-score normalization (scaling the data for each metabolite to have a mean of 0 and an SD of 1) for the PC plots was carried out in RStudio version 2025.09.2+418. The phylogenetic tree was drawn in MEGA11 (www.megasoftware.net) based on marker sequences publicly available on NCBI (see Rodriguez *et al*., 2009), using maximum-likelihood and a bootstrap value of 100.

## Results

### Experimental design

CBC metabolites were profiled in the apical leaf blade of the third fully expanded leaf, harvested under growth irradiance. For each species, 5–7 replicate samples were harvested from different plants. Metabolite levels were initially expressed on an FW basis. However, there can be systematic changes in the levels of all metabolites if leaves from different genotypes have differing water contents due, for example, to differing protein content and/or cytoplasmic volume and/or cell wall contribution. To exclude these secondary effects, in each sample the amount of C in a given metabolite was expressed as a percentage of the summed C in all measured metabolites (Supplementary Table S1). In previous publications (Arrivault *et al*., 2019) this normalization was termed a ‘dimensionless’ dataset. Xylulose 5-phosphate (Xu5P) and ribulose 5-phosphate (Ru5P) are not separated by the LC-MS/MS platform and are therefore represented together (Xu5P+Ru5P). Dihydroxyacetone phosphate (DHAP), which is the major triose phosphate, was detected, but glyceraldehyde-3-phosphate was not.

### Comparison of Calvin–Becson cycle metabolite levels in five different *Solanum* sect. *lycopersicon* species

**Fig. 1.**
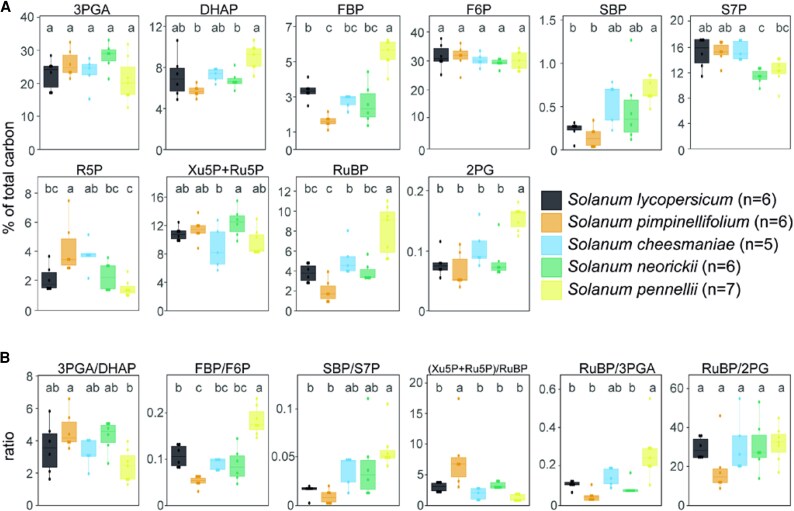
Levels of metabolites and ratios in five *Solanum* sect. *lycopersicon* species. (A) Metabolite contents. (B) Metabolite ratios. The 3PGA/DHAP ratio is a proxy for the balance between energy supply from the light reactions and energy consumption in the CBC. The FBP/F6P, SBP/S7P, and (Xu5P+Ru5P)/RuBP ratios are proxies for resistance to flux at FBPase, SBPase, and PRK, respectively. The RuBP/3PGA and RuBP/2PG ratios indicate resistance at the carboxylation and oxygenation reactions of Rubisco. Metabolite levels were normalized by expressing the C in a given metabolite as a percentage of the summed C in all CBC metabolites in that species (termed a ‘dimensionless’ dataset, see Arrivault *et al*., 2019). To do this, in a given sample, the level of each metabolite was first transformed to C equivalent values by multiplying the amount (nmol g FW^−1^) by the number of C atoms in the metabolite. The C equivalent amounts of all CBC intermediates plus 2PG were then summed for that sample. In the last step, the C equivalent value of a given metabolite was divided by the summed C equivalent value. Between five and seven replicate samples were harvested and analysed for each species. Single data points are shown on the graphs, the upper and lower limits of the boxes represent the third and first quartile, and the whiskers represent the interquartile range. The letters on the graph indicate significant differences between the means of each species, with *P*-value <0.05 (ANOVA and Tukey’s test performed in GraphPad Prism version 10.2.2, graphs generated in Rstudio version 2025.09.2+418). Note the different scales on the *y*-axes. Data and normalized data are provided in Supplementary Table S1, and non-normalized data are plotted in Supplementary Fig. S2.

The normalized metabolite levels in each genotype are shown as boxplots in Fig. 1A. A Tukey post-hoc test was performed to detect significative inter-species differences. There were many significant inter-species differences in the 10 pairwise comparisons, especially for ribulose 1,5-bisphosphate (RuBP; five), fructose 1,6-bisphosphate (FBP; five), sedoheptulose 7-phosphate (S7P; four), ribose 5-phosphate (R5P; four), and 2-phosphoglycolate (2PG; four), with fewer for DHAP (two), sedoheptulose 1,7-bisphosphate (SBP; two), and Ru5P+Xu5P (one), and none for 3PGA and fructose 6-phosphate (F6P) (Supplementary Table S1). Pairwise species comparisons pointed to *S. pennellii* being the most divergent species (in each comparison, 4–6 metabolites showed significant differences) followed by *S. pimpinellifolium* (1–6 metabolites) *S. cheesmaniae* (0–5 metabolites), *S. neorickii* (1–4 metabolites), and *S. lycopersicum* (0–4 metabolites). No significant differences were found between metabolites when *S. lycopersicum* and *S. cheesemaniae* were compared (Supplementary Table S1).

**Fig. 2.**
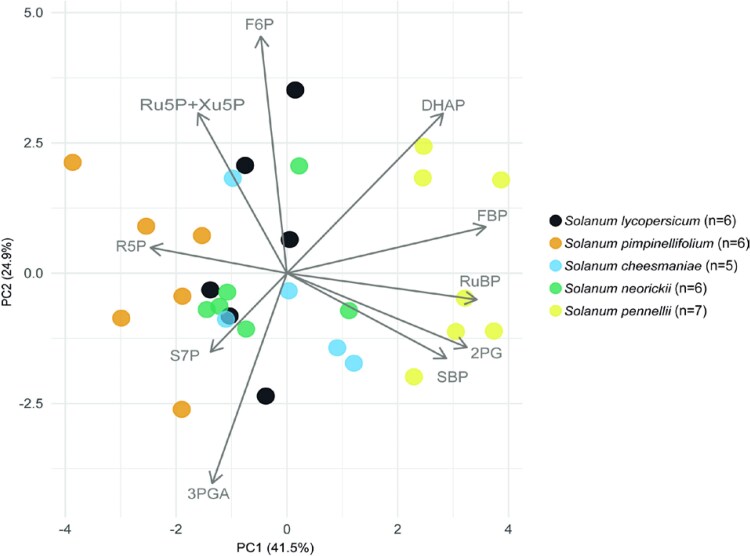
PC analysis of metabolite profiles from the five *Solanum* sect. *lycopersicon* species. Metabolite amounts were transformed into a dimensionless dataset (see legend of Fig. 1). Before the PC analysis, the values were *z*-score normalized, scaling the data for each metabolite to have a mean of 0 and an SD of 1. The *z*-score normalization and graph generation were carried out in RStudio version 2025.09.2+418. The display shows each replicate sample, and the vectors for CBC metabolites that drive separation of the samples.

To provide an integrated overview, PC analysis was performed on *z*-score-normalized data. PC1, PC2, and PC3 accounted for most of the total variation (41.5, 24.9, and 14.2%, respectively). The two major PCs are shown in Fig. 2. PC analysis separated *S. pennellii* from the other species. *Solanum pimpinellifolium* was also quite separated from other species, and took a diametrically opposite position to *S. pennellii*. The other three species overlapped with each other. Separation was mainly along PC1, driven by opposed changes of RuBP, SBP, FBP, 2PG, and DHAP, which aligned with *S. pennellii*, and R5P, Xu5P+Ru5P, S7P, and 3PGA, which aligned with *S. pimpinellifolium*. There was no evident separation of species in PC2.

### Comparison of metabolite ratios in five different *Solanum* sect. *lycopersicon* species

Metabolite ratios reflect the relative abundance of the substrate and product of a reaction, and provide a proxy for restriction on flux. The 3PGA/DHAP ratio (Fig. 1B), which is indicative of energy supply from the photosynthetic light reactions, was in the range expected in moderately light-limited photosynthesis. The only significant pair-wise difference was between a slightly lower 3PGA/DHAP ratio in *S. pennellii* and a slightly higher value in *S. pimpinellifolium*. Neither of these species was significantly different from the other three species. The FBP/F6P, SBP/S7P, and (Ru5P+Xu5P)/RuBP ratios (Fig. 1B) provide information for the reactions catalysed by fructose-1,6-bisphosphatase (FBPase), sedoheptulose-1,7-bisphosphatase (SBPase), and PRK, respectively. The FBP/F6P ratio was significantly higher in *S. pennellii* and lower in *S. pimpinellifolium* than in the other species. The SBP/S7P ratio was also significantly higher in *S. pennellii* than *S. pimpinellifolium*, and showed a similar but non-significant trend to the FBP/F6P ratio for the other three species. *Solanum pimpinellifolium* displayed a significantly higher (Ru5P+Xu5P)/RuBP ratio than the other four species. The RuBP/3PGA and RuBP/2PG ratios (Fig. 1B) reflect Rubisco carboxylation and oxygenation, respectively. *Solanum pennellii* showed a significantly higher RuBP/3PGA ratio than *S. lycopersicum*, *S.pimpinellifolium*, and *S. neorickii*, and a non-significant trend to a higher value than *S. cheesmaniae.* No significant differences were found for the RuBP/2PG ratio.

### Comparison of *Solanum* sect. *lycopersicon* species with phylogenetically distinct C_3_ species and C_4_ species

**Fig. 3.**
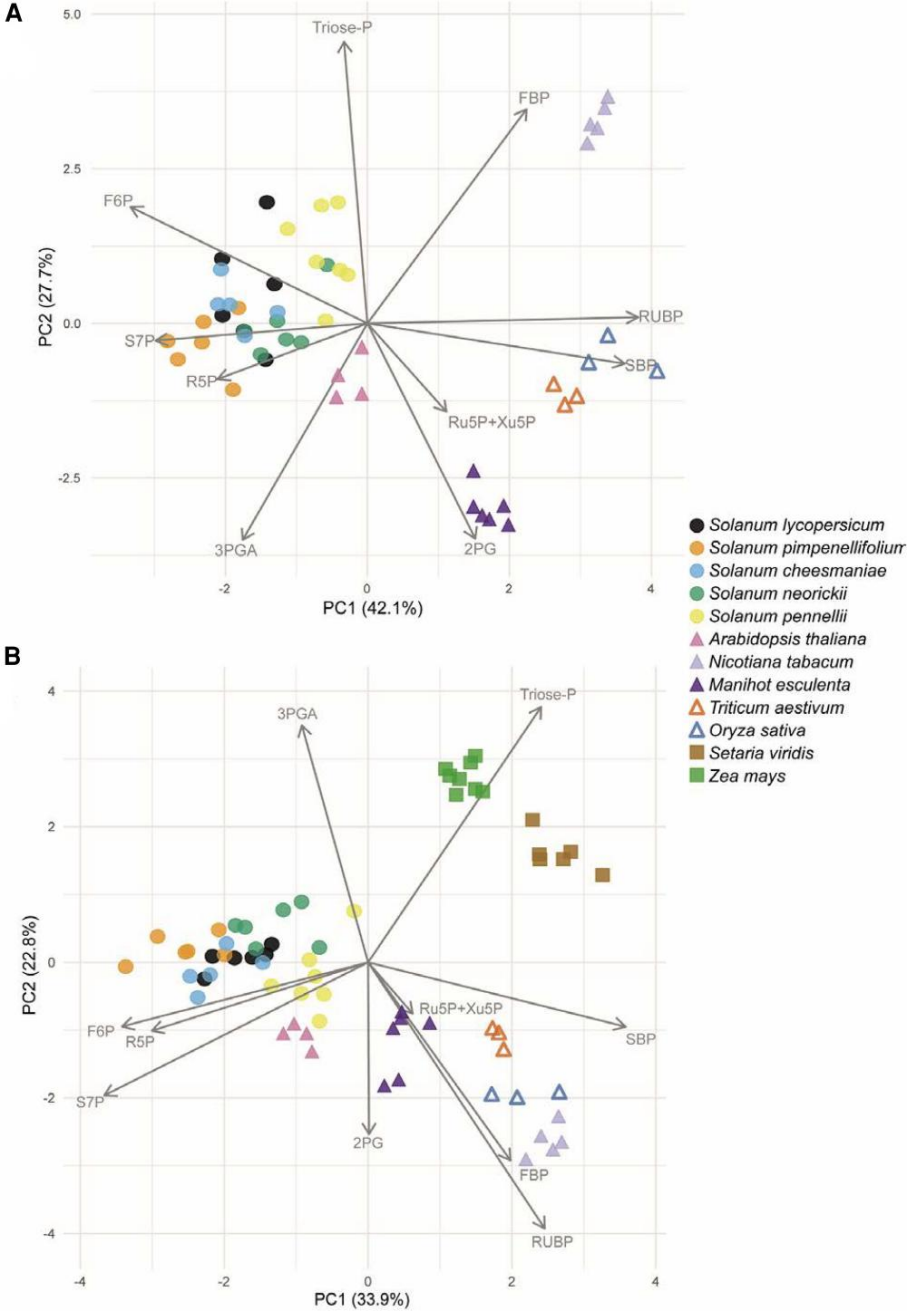
PC analysis of metabolite profiles from *Solanum* sect. *lycopersicon* species together with further published datasets. (A) Together with five further C_3_ species and (B) together with these five C_3_ species and two C_4_ species. Metabolite amounts were transformed into a dimensionless dataset for each sample (see legend to Fig. 1). Before the PC analysis, the values were *z*-score normalized (see legend to Fig. 2). The displays show each replicate sample, and the vectors for CBC metabolites that drive separation of the samples.

A second PC analysis was performed after combining the dataset for the five *Solanum* species with published datasets for five other C_3_ species (*A. thaliana*, *N. tabacum*, *O. sativa*, *T. aestivum*, and *M. esculenta*) from Arrivault *et al*. (2019). This analysis again used *z*-score-normalized data. PC1 and PC2, which accounted for 47.2% and 22.7% of total variation, respectively, are shown in Fig. 3A. The *Solanum* species, in particular *S. pennellii* and *S. pimpinellifolium*, remained partly separated from each other in this combined analysis. All of the *Solanum* species were clearly separated from the other C_3_ species. The closest was *A. thaliana*. whilst *T. aestivum*, *O. sativa*, *N. tabacum*, and *M. esculenta* were strongly separated from the *Solanum* species. The separation was seen mainly in PC1 and was driven by higher F6P, S7P, R5P, 3PGA, and to a certain extent DHAP, and lower RuBP, SBP, FBP, and to a certain extent 2PG and Xu5P+Ru5P in the *Solanum* species than the other C_3_ species. There was no consistent separation in PC2.

A third PC analysis was performed including the five *Solanum* species, the four above C_3_ species, and two C_4_ species (*Zea mays* and *Setaria viridis*) again using data from Arrivault *et al*. (2019). PC1 and PC2, which accounted for 36.2% and 25.4% of total variation, respectively, are shown in Fig. 3B. As seen in earlier studies (Arrivault *et al*., 2019; Borghi *et al*., 2022), C_3_ species separated from C_4_ species, driven mainly by higher 3PGA and/or DHAP levels in the C_4_ species. Even in this analysis of species with diverse modes of photosynthesis, the *Solanum* species are separated from other C_3_ species although again lying quite close to *A. thaliana*, and show partial separation between themselves.

## Discussion

The tomato *Solanum* sect. *lycopersicon* clade was used to test, firstly, if there is interspecies variation in CBC metabolite levels in a set of closely phylogenetically related species and, secondly, if the CBC metabolite profiles of this set of phylogenetically related species are distinct from or overlap with those of phylogenetically distant C_3_ species.

There were many significant differences in levels of individual CBC intermediates between the five *Solanum* species, especially for RuBP and FBP that showed five, and S7P and R5P that showed four significant differences in a total of 10 pair-wise comparisons (Fig. 1A; Supplementary Table S1). *Solanum pennelli* was the most divergent species, followed by *S. pimpinellifolium*, whilst *S. lycopersicum* ‘Moneymaker’ and *S. cheesmaniae* were the most similar. In a PC analysis of CBC metabolite profiles, *S. lycopersicum* ‘Moneymaker, *S. cheesmaniae*, and *S. neorickii* overlapped, and *S. pimpinellifolium* and especially *S. pennellii* grouped separately and were also at the opposite ends of the overall distribution (Fig. 2). These observations reveal variation in poising of the CBC between closely related species. As mentioned in the Introduction, environment influences intermediate levels. Our experiments in standardized conditions reveal the impact of genotype or, at least, a genotype×environment interaction. Our study complements other research that has identified variation in light acclimation, stomatal response, energy dissipation, and further photosynthetic traits between closely related or even within species (Jakobson *et al*., 2016; van Rooijen *et al*., 2017; Flood, 2019; Kromdijk and McCormick, 2022; Sakoda *et al*., 2022).

Separation of species in the PC analysis was mainly driven by opposing changes in RuBP, SBP, FBP, 2PG, and DHAP (higher in *S. pennellii*) and R5P, Xu5P+Ru5P, S7P, and 3PGA (higher in *S. pimpinellifolium*). To investigate which reactions might be responsible, metabolite ratios were calculated (Fig. 1B). The 3PGA/DHAP ratio did not change significantly except for somewhat lower values in *S. pennellii* compared with *S. pimpinellifolium*, indicating that the balance between energy provision by the electron transport chain and energy consumption in the CBC has shifted in favour of the former in *S. pennellii*. Within the CBC, *S. pennelli* was characterized by higher substrate/product ratios (indicating higher resistance to flux) at FBPase, SBPase, and Rubisco, whilst *S. pimpinellifolium* showed increased resistance to flux at PRK. The other species were intermediate between *S. pennellii* and *S. pimpinellifolium*. Lana-Costa *et al*. (2020) recently reported a quantitative trait locus (QTL) in an *S. pennelli* versus *S. lycopersicum* introgression line population that was associated with higher rates of photosynthesis and increased Rubisco activity. It is tempting to suggest that the higher FBP/F6P and SBP/S7P ratios and higher RuBP in *S. pennellii* in our study might reflect increased resistance to flux at FBPase and SBPase in *S. pennellii* and, possibly, increased binding of RuBP at more abundant Rubisco active sites.

Our observations are broadly consistent with the hypothesis that CBC metabolite profiles differ due to lineage-dependent adaptation in closely related C_3_ species, as the most divergent profiles were found in *S. pennelli*, which is more phylogenetically distant compared with the other species (Supplementary Fig. S1A). Within the group of more closely related species, the observed CBC metabolite profiles do not follow phylogeny so closely. In particular, the cultivated *S. lycopersicum* (variety Moneymaker) is very similar to *S. cheesmaniae* and *S. neorickii* but distinct from its progenitor *S. pimpinellifolium*. *Solanum pimpinellifolium* spans a long north–south axis along the Andes and has also spread from wet climate zones in coastal Ecuador to much drier zones in central and southern Peru (Lin *et al*., 2020; Supplementary Fig. S1B). Metabolic profiling of other sets of closely related species could reveal if diversity in CBC poising is a characteristic of only the *Solanum* clade, or also occurs in other groups of phylogenetically related species.

The *S. pimpinellifolium* accession used in this study (LA1589) has been described as endemic to central–south Peru (Lin *et al*., 2020). The modern tomato probably derived from more northerly populations (Lin *et al*., 2020), possibly explaining the metabolic space between *S. pimpinellifolium* and *S. lycopersicum*. Profiling of additional *S. pimpinellifolium* and *S. lycopersicum* varieties could ascertain whether the differences in CBC poise found in this study reflect genetic diversity of the starting breeding material or are a consequence of the breeding process itself, including effects of linkage drag. Reasons for the variance in CBC profiles between wild *Solanum* species also require further study. An attractive possibility is adaptation to differing ecological niches (Flood, 2019) including climatic factors such as aridity and altitude (Peralta *et al*., 2008; Pease *et al*., 2016; Gibson and Moyle, 2020; Lin *et al*., 2020). *Solanum pennellii* and *S. pimpinellifolium* have pronounced salinity tolerance (Bolger *et al*., 2014; Razali *et al*., 2018), which might be linked with the general metabolic landscape including CBC poise. Microclimate as a driver of (micro-) evolution has been discussed for *A. thaliana* (Hancock *et al*., 2011; Kubota *et al*., 2015). Another, and not mutually exclusive, possibility is that differences due to random genetic drift (Lynch *et al*., 2016) in common progenitors were exacerbated by geographical separation (Bock *et al*., 2023).

When the tomato clade is compared with other C_3_ species, their CBC metabolite profiles clustered together, separately from non-*Solanum* species. At the same time, variation can be seen in how the other four C_3_ species distribute. This probably points to a certain degree of conservation of CBC poise in *Solanum* species, which jointly share a different evolutionary trajectory from the other investigated C_3_ species. The dicot *A. thaliana* was most similar to the *Solanum* species, whilst the dicot species *N. tabacum* and *M. esculenta* and the monocot C_3_ species *O. sativa and T. aestivum* were much further separated from the *Solanum* sect. *lycopersicon* species. When the experimental space is expanded by including C_4_ species, the *Solanum* species still group separately from other C_3_ species. Overall, these observations are consistent with the idea that the CBC diverged in a phylogenetically related manner. However, even though *N. tabacum* is a member of the *Solanaceae* family, it grouped further away than *A. thaliana* from the *Solanum* species. This observation indicates that the extent of separation of CBC metabolite profiles is not solely due to phylogeny.

In conclusion, variation in CBC metabolite profiles within *Solanum*, together with their separation from other C_3_ and C_4_ species, indicates that CBC evolution is shaped by both phylogenetic relatedness and lineage-specific adaptation. Species variation in the *Solanum* could be further investigated using introgression lines of wild *Solanum* species (de Oliveira Silva *et al*., 2018; Lana-Costa *et al*., 2020). More generally, there is much interest in genetically improving photosynthesis (Raines, 2022; Long *et al*., 2025). Species variation in poising of the CBC should be taken into account when designing strategies to improve photosynthesis in crops.

## Supplementary Material

erag207_Supplementary_Data

## Data Availability

All data in this study are presented in the mansucript and its Supplementary data available online.

## Supplementary data

The following supplementary data are available at *JXB* online.

Fig. S1. Tomato clade (*Solanum* sect. *lycopersicon*) phylogenetic tree and geographical distribution of wild species.

Fig. S2. Levels of CBC intermediates and 2PG.

Table S1. Content of CBC intermediates and 2PG in species of *Solanum* sect. *lycopersicon* and pair-wise species comparison of metabolite levels.

## Abbreviations

CBC: Calvin–Benson cycle
DHAP: dihydroxyacetone phosphate
F6P: fructose 6-phosphate
FBP: fructose 1,6-bisphosphate
FBPase: fructose-1,6-bisphosphatase
PC: principal component
2PG: 2-phosphoglycolate
3PGA: 3-phosphoglycerate
PRK: phosphoribulokinase
R5P: ribose 5-phosphate
Ru5P: ribulose 5-phosphate
RuBP: ribulose 1,5-bisphosphate
S7P: sedoheptulose 7-phosphate
SBP: sedoheptulose 1,7-bisphosphate
SBPase: sedoheptulose-1,7-bisphosphatase
Xu5P: xylulose 5-phosphate
