# Supplementary material for: Variance in Calvin–Benson cycle intermediate levels between closely related species in the tomato clade
Source: J Exp Bot. 2026 May 2;77(14):4392–400. doi: 10.1093/jxb/erag207 (PMC13415956; doi:10.1093/jxb/erag207)

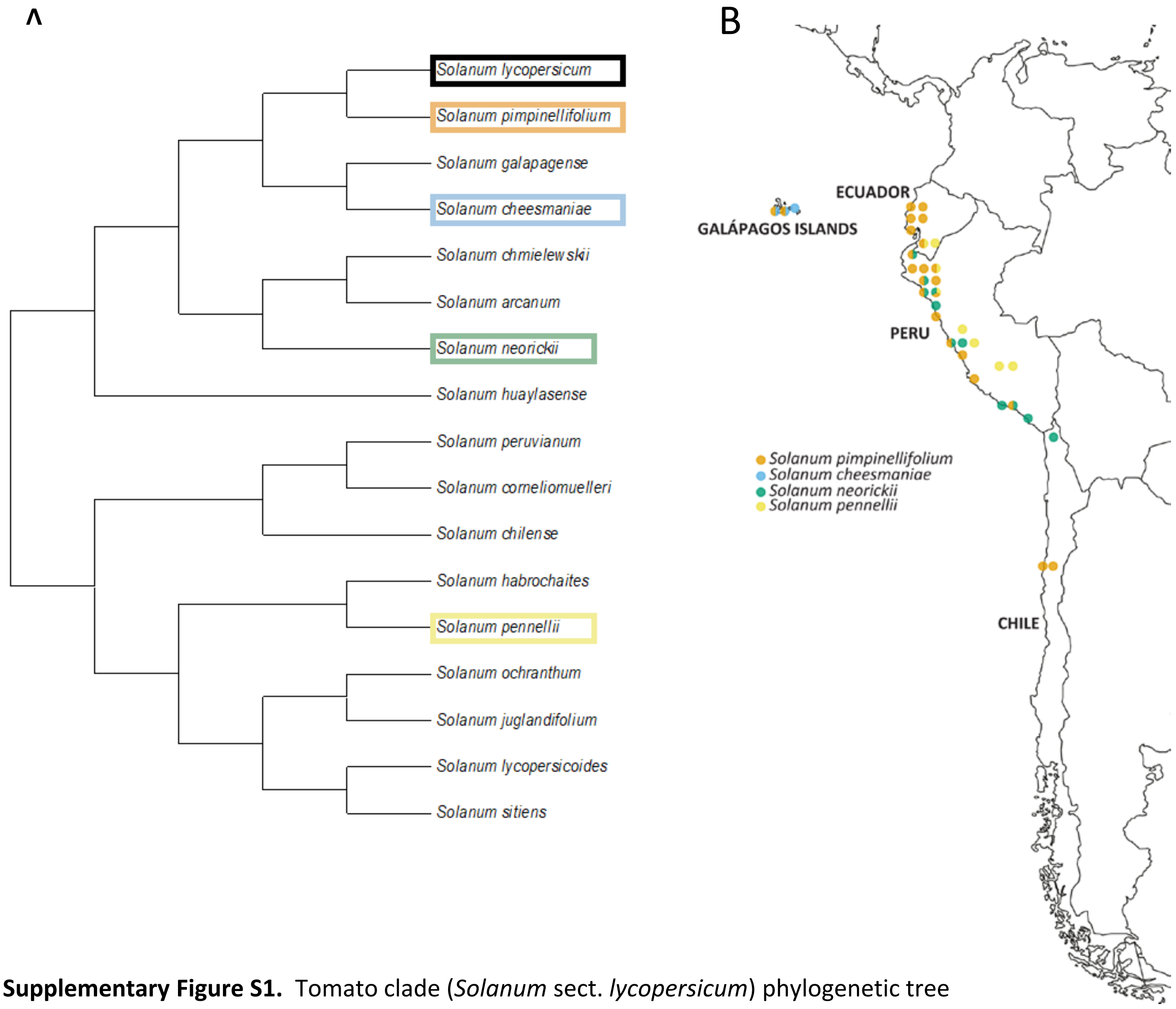

Supplementary Figure S1. Tomato clade (*Solanum* sect. *lycopersicum*) phylogenetic tree and geographical distribution of wild species.

(A) Phylogenetic tree, based on a combined analysis of 18 COSII (conserved orthologous set nuclear loci) markers (as in Rodriguez et al., 2009). The species included in the experiments are indicated by coloured boxes. The phylogram also includes broad-sense relatives of tomato (sister groups) *S. ochranthum*, *S. juglandifolium*, *S. lycopersicoides* and *S. sitiens*. The phylogenetic tree was drawn in MEGA11 (www.megasoftware.net) based marker sequences publicly available on NCBI (see Rodriguez et al., 2009), using maximum-likelihood and a bootstrap value of 100.

(B) Geographical distribution of wild tomato relatives, drawn based on data from Peralta *et al*., (2008).
Solanum pimpinellifolium is native to coastal areas up to 500 m above sea level with a wide distribution from central coastal Equador and northern Peru through to central Chile. It is found mainly in wet places like river valleys that receive seasonal water from the Andes, and on the edges of cultivated fields where it exposed to high salinity. In the Galápagos Islands, where *S. pimpinellifolium* has been introduced or arrived recently, it forms hybrids with the endemic Galápagos tomato *S. cheesmaniae. S. pimpinellifolium* is very closely related to the cultivated tomato S. *lycopersicum* and hybridizes with it freely. *S. pimpinellifolium* requires moisture, like S. *lycopersicum* .
Solanum cheesmaniae is found on the Galápagos Islands, in dry rocky areas from sea level up to volcanic peaks 1300 m above sea level.
Solanum neorickii is found in Southern Peru to southern Ecuador, in dry valleys in the very dry high-altitude deserts on the western slopes of the Andes, 1950-3000 m above sea level, often found trailing over rocky banks and roadsides.
Solanum pennellii is a spreading perennial herb found in Northern Peru to northern Chile. It grows on dry rocky hillsides and sandy areas along the western Andean slopes up to 3000 m above sea level. It is also found in the lomas, small areas of vegetation near the coast where moisture is provided by fog from the ocean, in an otherwise extremely dry desert.

Supplementary Table S1: Content of CBC intermediates and 2PG in species of *Solanum* sect. *Lycopersicon,* and pair-wise species comparison of metabolite levels. The upper left Table shows amounts and the upper right Table shows data after normalisation to remove compounding effects of change in leaf protein or cell wall content or vacuole size: the level of each metabolite was transformed to C equivalent values by multiplying the amount (nmol g FW^{-1}) by the number of C atoms in the metabolite, the C equivalent amounts of all CBC intermediates plus 2PG were summed for that sample, and the C equivalent value of a given metabolite was divided by the summed C equivalent value for all measured metabolites. The bottom Table shows pairwise species comparison of metabolites levels with Tukey post-hoc test p-values. The analyses were performed on the 'dimensionless' data set, i.e. after expressing the C in a given metabolite as a % of the total C in measured metabolites in that species. Significant differences between species (with p-value < 0.05) are highlighted in red. The number of significant changes between species in a given pair-wise comparison is presented in the last (right hand) column. A total of was 7, 8, 7, 11 and 17 significant changes were detected in the pairwise comparisons of *S. lycopersicum*, S. *neorickii, S. pimpinellifolium*, *S. cheesmaniae* and *S. pennelli* with other species, respectively. The number of significant changes in the levels of a given metabolite across pairwise comparisons is given in the bottom row. Non-normalised data are plotted in Supplementary Figure S1, and normalised data in Figure 1

	Content (nmol/g FW)										Normalised as a % of total carbon measured metabolites in the sample									
	RuBP	3PGA	DHAP	FBP	F6P	SBP	S7P	R5P	2PG	Ru5P+Xu5P	RuBP	3PGA	DHAP	FBP	F6P	SBP	S7P	R5P	2PG	Ru5P+Xu5P
S. lycopersicum	45.1	430.4	73.9	25.4	191	2	113	14.2	1.8	89.2	5.2	29.6	5.1	3.5	26.3	0.3	18.1	1.6	0.1	10.2
S. lycopersicum	27.3	422.1	106.4	29.7	249.6	0.3	121.6	28.2	1.1	100.4	2.8	25.9	6.5	3.6	30.6	0	17.4	2.9	0.1	10.2
S. lycopersicum	47.9	408.4	250.1	36.8	443.3	2.2	115.3	22.1	1.3	175.8	3.5	17.7	10.9	3.2	38.5	0.2	11.7	1.6	0.1	12.7
S. lycopersicum	31.6	306.1	145.6	29.6	317.2	2	115.2	40.3	1.3	122.4	3	17.6	8.3	3.4	36.4	0.3	15.4	3.8	0.1	11.7
S. lycopersicum	40.1	402.3	87.7	19.9	240.3	1.9	119.4	14.1	1.3	100.6	4.3	25.9	5.6	2.6	30.9	0.3	17.9	1.5	0.1	10.8
S. lycopersicum	52.6	441.9	139.8	39.1	298.7	2.1	106.4	23.6	1.5	124.9	4.7	23.9	7.6	4.2	32.3	0.3	13.4	2.1	0.1	11.3
S. neorickii	35.3	520.9	119	18.5	263.2	1.3	98.2	33	1.5	104.8	3.4	30.2	6.9	2.1	30.5	0.2	13.3	3.2	0.1	10.1
S. neorickii	43.9	658.4	131.3	15.6	330.4	6.2	122.5	42.2	1.6	176.2	3.3	29.6	5.9	1.4	29.8	0.6	12.9	3.2	0.1	13.2
S. neorickii	38.3	564.4	118.4	16.4	269.7	3.3	90.7	16.4	1.4	150.7	3.5	31	6.5	1.8	29.7	0.4	11.6	1.5	0.1	13.8
S. neorickii	64.9	814.3	159.8	31.5	327.2	3.1	113.7	19.9	1.7	179.8	4.5	33.8	6.6	2.6	27.2	0.3	11	1.4	0.1	12.5
S. neorickii	59.9	375.1	144.4	38.9	269.2	0.9	71.6	15.8	1.1	163	5.9	22	8.5	4.6	31.6	0.1	9.8	1.5	0.1	15.9
S. neorickii	40	523.2	140.5	33.9	304.8	10.9	98	43.6	2.9	128.8	3.4	26.8	7.2	3.5	31.2	1.3	11.7	3.7	0.1	11
S. pimpinellifolium	63.2	618.8	175.3	28.9	433.7	2.3	168.3	45.8	1.4	191.7	4	23.6	6.7	2.2	33.1	0.2	15	2.9	0.1	12.2
S. pimpinellifolium	26.6	598.7	153	21.3	427.3	0.6	166.3	55.1	1.4	183	1.8	24.1	6.2	1.7	34.4	0.1	15.6	3.7	0.1	12.3
S. pimpinellifolium	41	804.8	122.2	17.8	300.7	3.6	177.8	44.9	2.4	163	2.8	33.5	5.1	1.5	25	0.4	17.3	3.1	0.1	11.3
S. pimpinellifolium	15.7	704.7	179.1	18.2	592.8	0.7	173.7	148.9	1.3	272.6	0.8	21.7	5.5	1.1	36.6	0	12.5	7.7	0	14
S. pimpinellifolium	28.8	750.8	170.5	21.9	458.4	2.7	211.5	59.5	3.2	190.7	1.7	26.3	6	1.5	32.2	0.2	17.3	3.5	0.1	11.2
S. pimpinellifolium	19.5	885.7	163.2	26.7	452.1	0.2	196.3	96.6	1.6	158.3	1.1	30.3	5.6	1.8	30.9	0	15.7	5.5	0.1	9
S. pennellii	80.2	278.9	102.5	38.7	178.3	4.7	76.9	8.4	2.1	62.3	10.6	22.1	8.1	6.1	28.3	0.9	14.2	1.1	0.2	8.2
S. pennellii	40.8	220.5	130.5	33.3	226.4	3.4	69.4	21.3	2.4	87.4	5.3	17.1	10.1	5.1	35.1	0.6	12.5	2.7	0.2	11.3
S. pennellii	45.2	194.1	125.6	33.4	192.2	2.4	57.8	10.5	1.5	91.3	6.5	16.8	10.8	5.8	33.2	0.5	11.6	1.5	0.1	13.1
S. pennellii	83.1	413.1	104.4	29.3	189	4	77.2	16.4	2.4	74.8	9.6	28.8	7.3	4.1	26.3	0.6	12.5	1.9	0.2	8.7
S. pennellii	36.3	362	105.4	35.4	153.4	4.2	40.5	6.9	1.5	58	5.4	32.3	9.4	6.3	27.4	0.9	8.4	1	0.1	8.6
S. pennellii	68.6	131.7	102	32.2	171.3	2.8	58.4	8.4	1.5	64.5	11.2	12.9	10	6.3	33.6	0.6	13.4	1.4	0.1	10.5
S. pennellii	57.6	212.8	86.9	27.4	158.8	3.5	65.2	8.1	1.8	52	9.3	20.7	8.4	5.3	30.8	0.8	14.8	1.3	0.2	8.4
S. cheesmaniae	107.2	572.6	141.6	33.8	334	6.6	135.4	29.7	2	87.2	8.3	26.6	6.6	3.1	31	0.7	14.6	2.3	0.1	6.7
S. cheesmaniae	63.2	477.4	151.9	28.7	283.3	6.5	141	44.7	3.1	66.4	5.6	25.2	8	3	30	0.8	17.4	3.9	0.2	5.8
S. cheesmaniae	43.9	414.5	136.5	28	288	5.5	118.2	57.7	2.1	90.4	4.1	23.1	7.6	3.1	32.1	0.7	15.4	5.4	0.1	8.4
S. cheesmaniae	45	252.9	128.9	20.9	276.7	1.6	121.9	36.7	1.3	126.2	4.7	15.7	8	2.6	34.3	0.2	17.6	3.8	0.1	13
S. cheesmaniae	61.1	700.4	174.6	27.2	347.4	3.8	153.4	60.3	2.3	170.1	4.1	28.3	7	2.2	28	0.4	14.4	4.1	0.1	11.4

	Pairwise species comparison of normalised metabolite levels<br>Tukey post-hoc comparison p-values										Number of metabolites showing significant changes between species (maximum = 10)
	RuBP	3PGA	DHAP	FBP	F6P	S7P	R5P	Xu5P+Ru5P	SBP	2PG	
S. lycopersicum* vs. *S. neorickii	>0.999	0.366	0.983	0.426	0.730	0.013	0.999	0.628	0.369	0.996	**1**
S. lycopersicum* vs. *S. pimpinellifolium	0.3114	0.803	0.288	0.003	>0.999	>0.999	0.029	0.987	0.973	0.954	**2**
S. lycopersicum* vs. *S. pennelli	0.0004	0.978	0.078	0.000	0.925	0.061	0.835	0.811	0.014	0.000	**4**
S. lycopersicum* vs. *S. cheesmaniae	0.6032	>0.999	1.000	0.672	0.964	0.999	0.180	0.464	0.204	0.432	**0**
S. neorickii* vs. *S. pimpinellifolium	0.2654	0.942	0.581	0.157	0.803	0.013	0.048	0.890	0.133	0.826	**2**
S. neorickii* vs. *S. pennelli	0.0006	0.123	0.023	<0.001	0.989	0.923	0.707	0.111	0.520	0.001	**4**
S. neorickii* vs. *S. cheesmaniae	0.6624	0.488	0.961	0.997	0.984	0.011	0.262	0.041	0.990	0.636	**2**
S. pimpinellifolium* vs. *S. pennelli	<0.001	0.441	0.001	<0.001	0.961	0.061	0.002	0.507	0.003	<0.0001	**6**
S. pimpinellifolium* vs. *S. cheesmaniae	0.0210	0.886	0.258	0.103	0.984	0.999	0.944	0.229	0.067	0.147	**1**
S. pennelli* vs. *S. cheesmaniae	0.0320	0.959	0.141	<0.001	>0.999	0.048	0.019	0.954	0.840	0.037	**4**
Number of significant changes for that metabolite in pairwise species comparisons (maximum = 10)	**5**	**0**	**2**	**5**	**0**	**4**	**4**	**1**	**2**	**3**	

Supplementary Figure S2: Levels of CBC intermediates and 2PG. Between 5 and 7 replicate samples were harvested and analysed for each species. Single datapoints are shown on the graphs, the upper and lower limits of the boxes represent the third and first quartile, and the whiskers represent the interquartile range. The letters on the graph indicate significant differences between the means of each species, with p-value < 0.05 (ANOVA and Tukey's test performed in GraphPad Prism version 10.2.2, graphs generated in Rstudio version 2025.09.2+418). In Figure 1, the data is plotted after normalisation to show the C in each metabolite as a % of total C in CBC intermediates, and with the y-axis scale adjusted to display the data.

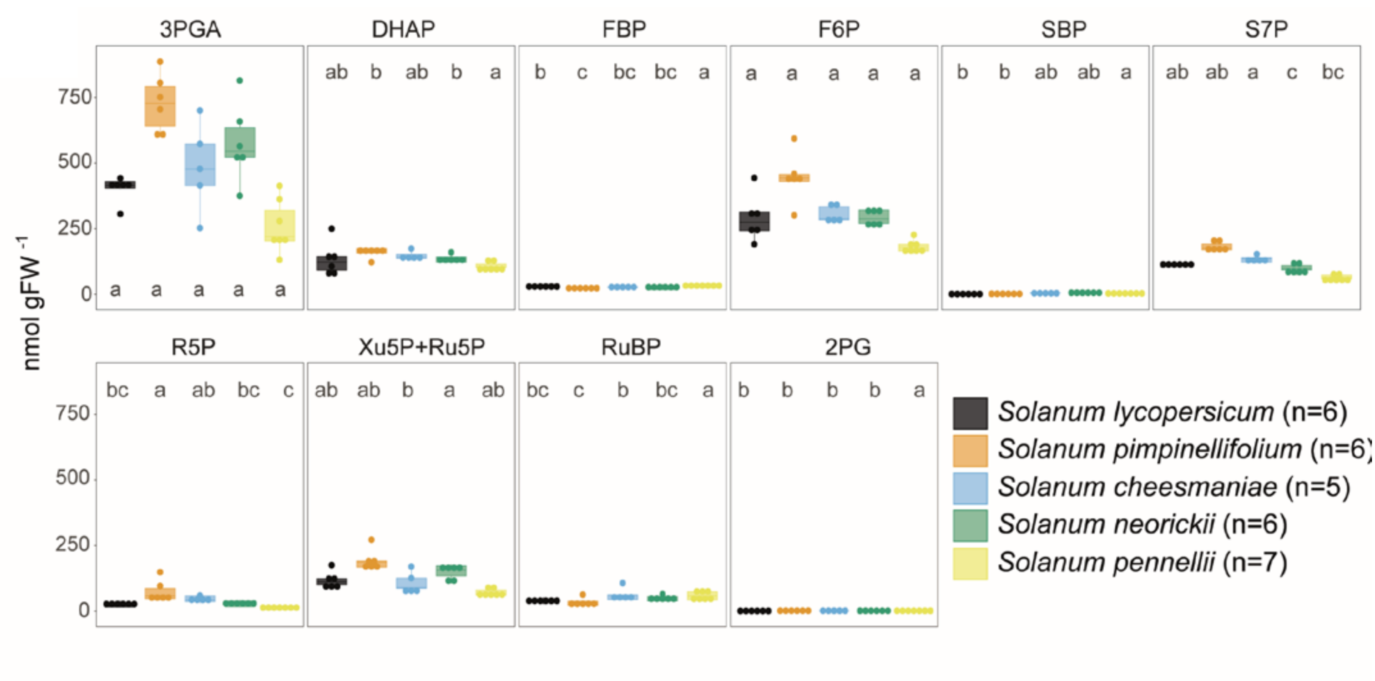

Supplement: erag207_Supplementary_Data [file erag207_supplementary_data.pdf]
